# Acceptability, feasibility and short-term outcomes of temperament based therapy with support (TBT-S): a novel 5-day treatment for eating disorders

**DOI:** 10.1186/s40337-023-00878-w

**Published:** 2023-09-14

**Authors:** Kristin Stedal, Ingrid Funderud, Christina E. Wierenga, Stephanie Knatz-Peck, Laura Hill

**Affiliations:** 1 Division of Mental Health and Addiction, Regional Department for Eating Disorders, Oslo University Hospital, Ullevål HF, Oslo, Norway; 2 Department of Psychiatry, Eating Disorder Treatment and Research Center, University of California San Diego, San Diego, CA USA; 3 Department of Psychiatry and Behavioral Health, The Ohio State University, Ohio, OH USA

**Keywords:** Anorexia nervosa, Bulimia nervosa, Eating disorders, Family-based treatment, Treatment, Temperament based treatment

## Abstract

**Background:**

Temperament Based Therapy with Support (TBT-S) aims to target the mechanisms underlying the aetiology and maintenance of eating disorders, and was developed as an adjunct to treatment as usual. There is limited research investigating acceptability, feasibility and possible benefits of TBT-S. Therefore, the primary aim of the current study was to assess treatment feasibility and acceptability at a tertiary specialized eating disorders service in Norway, with a secondary aim to explore possible benefits in clinical outcome.

**Methods:**

Forty-one patients (mean age 25.3, range 18–43) and 58 supports were assessed pre- and post TBT-S. The majority of the patients were diagnosed with either anorexia nervosa or atypical anorexia nervosa. Participants completed an 18-item Patient and Support Satisfaction Questionnaire, in addition to a questionnaire assessing the usefulness of the different intervention components and strategies utilised in TBT-S, as well as a 4-item treatment satisfaction questionnaire. Measures of treatment efficacy were completed at both time-points, whereas treatment acceptability was only assessed post-treatment.

**Results:**

Findings reveal that TBT-S is a feasible treatment with high client satisfaction. Preliminary outcome data were also encouraging, and in line with previous studies. There were no voluntary drop-outs. All participants, both patients and supports, reported that TBT-S helped them deal more effectively with their challenges. After completing treatment, there was a significant decrease in patients’ self-reported eating disorder psychopathology, psychosocial impairment and state anxiety, while trait anxiety remained unchanged. Patients also reported significantly improved social relationships, whereas supports reported a significant increase in (own) psychological health. There were no differences in family functioning.

**Conclusions:**

TBT-S is a promising new treatment for eating disorders with high acceptability scores and low treatment attrition. Future studies should aim to explore methods which can most appropriately measure the effect of TBT-S and the usefulness of the different components of this treatment. Randomised controlled trials are needed to assess treatment efficacy of TBT-S.

**Supplementary Information:**

The online version contains supplementary material available at 10.1186/s40337-023-00878-w.

## Introduction

Eating Disorders are disabling mental health disorders, associated with substantial functional impairment in occupational, social, and family domains. It has been estimated that it causes the loss of 3.3 million healthy life years every year [1], and for two of the eating disorders, anorexia nervosa (AN) and bulimia nervosa (BN), the mortality risks are about twice those of controls when treated outside the hospital [2]. Further, despite advances in treatments for BN (e.g. cognitive behaviour therapy, CBT), there is a paucity of evidence-based approaches for AN—in particular for the severe and enduring cases [3, 4].

For young patients, family based treatment is considered the gold-standard intervention [5]. However, it is not yet well understood how to best treat adult patients with AN, and a substantial proportion of patients do not respond to treatment, or experience relapse [6]. In an effort to more directly target the mechanisms underlying the aetiology and maintenance of eating disorders, Temperament Based Therapy with Support (TBT-S) was developed as an adjunct to treatment as usual [7]. TBT-S builds on research from multi-family treatments for eating disorders, and combines this with empirically based biological models (see Additional file 1 for Chapter 1 of the treatment manual [7] and a detailed overview of TBT-S). Although elements and some modules of TBT-S have previously been investigated and proposed as useful for patients with AN, BN and avoidant/restrictive food intake disorder (e.g. see [8–12]), the acceptability, feasibility and possible benefits of the full TBT-S treatment model has limited research. In addition, there have not been any studies conducted (1) in a different health-care system than the treatment's origins and (2) by a different clinical team than the treatment originators and finally (3), based on the newly published TBT-S treatment manual [7]. Thus, given the relative novelty of TBT-S, there is a need for studies examining the acceptability and feasibility of this approach—and to explore how it applies across cultures and health-care settings. Therefore, the primary aim of the current study was to assess treatment feasibility and acceptability at a tertiary specialized eating disorders service in Norway, with a secondary aim to explore possible benefits in clinical outcome.

## Methods

### Design

The current study was naturalistic, with an uncontrolled design and explorative aim. Since October 2020 up until March 2023, twelve groups, and a total of 45 patients and 65 supports have received the full 5-day TBT-S treatment at a tertiary specialised eating disorders service in Norway. Patients and supports who consented to be included in the study were assessed pre- and post-treatment. Efficacy measures were completed at both time-points, whereas treatment acceptability was only assessed post-treatment.

### Intervention

Adult TBT-S treatment was developed by Dr. Laura Hill together with clinicians and researchers at the University of California San Diego. The treatment strategies of TBT-S have been developed and adapted over a 10-year period through iteratively integrating client and support feedback with research findings from neurobiological studies. The treatment development process has spanned over a decade to increase accuracy and acceptability. Continuous advice from users have helped improve and structure the treatment based on their feedback. The Norwegian version of TBT-S was first implemented at the out-patient unit of the Regional Department for Eating Disorders (RASP) at Oslo University Hospital Ullevål in 2020. Under the supervision of one of the TBT-S developers (LH) we adopted the treatment structure for adult patients as described in the manual [7]. This structure consists of five consecutive days of treatment, up to eight hours each day. In accordance with the manual, TBT-S was delivered in a multi-client and support (e.g. parent(s), partner, other family member and/or friend) format and we utilized all the different intervention strategies to apply TBT-S principles, including: (1) neurobiological psychoeducation; (2) experiential learning activities addressing neurobiology and traits (3) client and support skills training; (4) meal coaching, and (5) the TBT-S behavioural agreement [7]. The clinical team received regular supervision and assistance from the TBT-S developers and the translation of materials from English to Norwegian was done in close collaboration with the treatment model developers. The TBT-S treatment was delivered by a clinical team consisting of one clinical psychologist, one psychiatric nurse, two registered dieticians and one psychologist/researcher. The full clinical team was present during most parts of the treatment—even when they did not have an active role in the intervention strategy being delivered.

### Inclusion procedures

RASP is a third line service for patients with eating disorders. Eligible patients were referred to TBT-S by their local treatment provider as a 5-day add-on to treatment as usual. Within one to two weeks, all referrals were reviewed by the RASP clinical review board. After approval by the board, referrals were sent to the TBT-S clinical team. One or two members of the TBT-S clinical team then conducted a one hour consultation with the patient and his/her support(s). This consultation had two main purposes; (1) to assess the patients’ and supports’ eligibility to be included in TBT-S, and (2) to provide an opportunity for potential participants to meet representatives from the clinical team and to address any questions or concerns regarding the TBT-S week. Criteria for inclusion to the TBT-S program were (i) a primary eating disorder diagnosis; (ii) medically stable as assessed through review of relevant medical information provided by referring clinician; (iii) BMI > 15; (iv) a minimum of one support who could participate throughout the full TBT-S week; (v) the patient had traits common to AN, e.g. anxiety, high attention to detail, altered interoceptive awareness, and (vi) willingness to participate in group activities. No other exclusion criteria were applied. Within one week, patients were notified about acceptance to the TBT-S treatment program. Consultations were done consecutively as soon as the referrals were forwarded by the review board. Thus, time between consultation and TBT-S week would vary greatly, from one week to three months, depending on the next available treatment spot. All patients received a telephone reminder one week before treatment week commenced. During this phone call, patients and supports were informed about the possibility to participate in our feasibility study. Participants who agreed to receive more information about the study were sent an email with a link to the secure data collection system nettskjema.no, a survey solution developed and hosted by the University of Oslo (nettskjema@usit.uio.no). Patients and supports who consented to participate were enrolled into the study.

## Measures

### Acceptability and feasibility measures

The 18-item TBT-S Patient and Support Satisfaction Questionnaire (PSSQ; [11]) was completed at the end of treatment and was employed to assess degree of satisfaction with the overall program, as well as some of the specific elements of TBT-S. This questionnaire has been used in a previous study of TBT-S [11] and thereby allows for comparisons of acceptability across health-care systems and cultures. Satisfaction is rated on a 5-point Likert scale (1 = Strongly Disagree to 5 = Strongly Agree). Chronbach’s alpha for the PSSQ in the current study was high for both patients (0.847) and supports (0.848). Participants also completed an 18-item questionnaire specifically assessing the helpfulness and usefulness of the different intervention components and strategies utilised in TBT-S (TBT-S Comp), including mealtimes, patient groups, dietician groups, neurobiological psychoeducation etc. Component usefulness was rated on a 10-point scale (1 = not at all helpful to 10 = very helpful). We also asked the participants four questions to rate how satisfied they were with the treatment program to assess the patients’ and supports’ treatment satisfaction with TBT-S (TBT-S Program Satisfaction Questionnaire, TBT-S SQ). The questions were answered on a 4-point scale, with higher scores indicating more satisfaction. Supports were asked if they had previously been included in treatment and if they wished they had been included more in previous treatments. Daily attendance was recorded. Treatment drop-out was the primary measurement of feasibility and was defined as premature cessation of treatment before completing the full TBT-S treatment week. A daily feedback form was administered for the participants to provide day-by-day evaluations and was also used to track dropout.

### Clinical measures

Patients’ height and weight was measured by a TBT-S clinician immediately prior to starting the treatment week and on the last day of the treatment. Diagnoses were set by a clinician with over a decade of experience working with eating disorders. The self-report assessments were completed within three days before treatment and immediately after, by both patients and supports. The pre-assessment included demographic information and information on (for patients) current treatment status and history, duration of illness and medication use.

The Eating Disorders Examination Questionnaire (EDE-Q, [13, 14]) is an assessment tool used to assess the presence and severity of ED symptoms during the previous 28 days. Supports were administered the parent version (P-EDEQ, [15]). The EDE-Q consists of 28 items measuring four clinically derived subscales (Restraint, Eating Concern, Shape Concern and Weight Concern) and a global score. Scores range from 0 to 6, with higher scores reflecting greater pathology. A modified version was used at discharge due to the short duration of the TBT-S program [10]. The Norwegian version of the EDE-Q has been shown to have reasonable psychometric properties and validity [16, 17], with a clinical cut-off score of 2.09 for AN [18].

The McMaster Family Assessment Device (FAD, [19]) is a questionnaire assessing family functioning, including patterns of transactions as well as organizational and structural components. The FAD consists of seven scales, which measure Problem Solving, Communication, Roles, Affective Responsiveness, Affective Involvement, Behavior Control and General Functioning. Scores range from 1 (healthy functioning) to 4 (unhealthy functioning), with higher scores in the General Functioning indicating worse level of family function. Both patients and supports were administered the FAD and scores on General Functioning were calculated. An approved Norwegian version of the FAD was employed (anne.m.sund@ntnu.no).

The World Health Organization’s Quality of Life Questionnaire (WHOQOL-Bref [20]) was also administered to both patients and supports. It is a frequently used instrument to assess the quality of life in both healthy and ill populations. The Norwegian version of the WHOQOL-Bref has demonstrated acceptable psychometric properties [21] and validity [22].

Finally, patients completed the The Spielberger State-Trait Anxiety Inventory (STAI, [23]) and the Clinical Impairment Assessment (CIA, [24]). The STAI is a 40-item questionnaire assessing the presence of anxiety. Each item is rated on a four-point scale, ranging from 1 to 4. Higher scores indicate higher anxiety. The STAI consists of two subscales, one assessing state anxiety and the other assessing trait anxiety. A Norwegian version of the STAI, with acceptable psychometric properties, was administered [25]. The CIA is a 16-item self-report measure of the psychosocial impairment caused by the eating disorder in the last 28 days. This measure was modified at post-assessment to reflect the last 7 days. The CIA provides a global score as well as three subscale scores (Personal Impairment, Social Impairment and Cognitive Impairment). The CIA aims to measure the severity of the eating disorders’ impact on psychosocial and physical functioning of the individual. Higher scores indicate greater severity. The Norwegian version has demonstrated satisfactory psychometric properties [26], and a clinical cut-off score of 16.0 [27].

### Ethics

The study was approved by the Regional Committee for Medical and Health Research Ethics (69871/REK sør-øst). Data were collected and stored on the TSD (Tjeneste for Sensitive Data) facilities, owned by the University of Oslo, operated and developed by the TSD service group at the University of Oslo, IT-Department (USIT).

## Analyses

### Descriptive statistics

For treatment acceptability, as measured by the 18-item PSSQ and the TBT-S Comp, we calculated group mean scores for patients and supports separately. The treatment satisfaction questions (TBT-SQ) were assessed by calculating the percentage of participants who selected each of the options on the 4-point scale. Treatment feasibility was examined by tallying treatment dropouts. Within-subject changes in clinical measures between pre- and post-treatment were analysed using paired samples t-tests using IBM SPSS Statistics for Windows, Version 28.0. The analysis of P-EDEQ was weighted for number of supports per patient.

## Results

Forty-one patients (mean age 25.3, range 18–43) and 58 supports were included in the current study. Forty of the supports were the patient’s parents (25 mothers, 15 fathers), eight were a married partner or cohabitant, and one was non-cohabitant partner. Based on ICD-10 criteria [28] the majority of the patients were diagnosed with either AN (n = 13, 32%) or atypical AN (n = 22, 54%). See Table 1 for patient descriptives. Forty-two percent of the supports reported that they had previously been included in treatment and 65% wished they had been more involved in previous treatments.

**Table 1:** Patient descriptives

	n	Pre-treatment mean (*SD*). range
Age (years)	41	25.3 (6.5), 18–43
BMI (kg/m^2^)	38	19.2 (*2.9*)_*3*_ 15.4–31.1
Duration of Illness (months)^a^	25	94.9 (87.2), 9–360
*Eating disorder diagnosis (ICD-10)*	41	n (%)
Anorexia nervosa		13 (32%)
Atypical anorexia nervosa		22 (54%)
Bulimia nervosa		3 (7%)
Unspecified eating disorder		3 (7%)
*Current treatment status*^a^	36	
In-patient treatment		2 (6%)
Day-patient treatment		6 (17%)
Out-patient		25 (69%)
No treatment		3 (8%)
Other		0 (0%)

*SD* standard deviation, *BMI* body mass index

^a^Self-reported

### Feasibility and acceptability

**Fig. 1:**
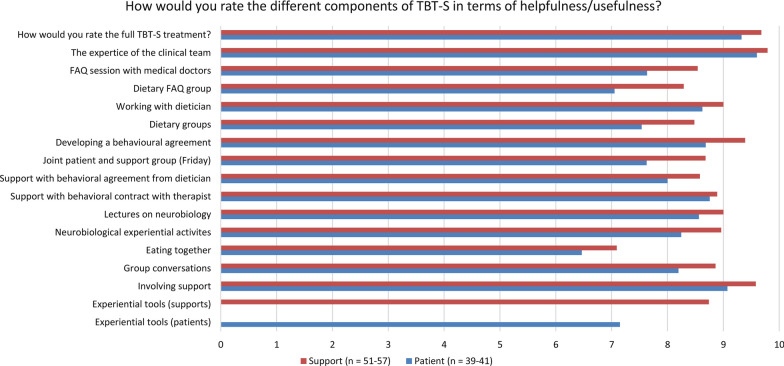
TBT-S post treatment components assessments (TBT-S Comp)

**Fig. 2:**
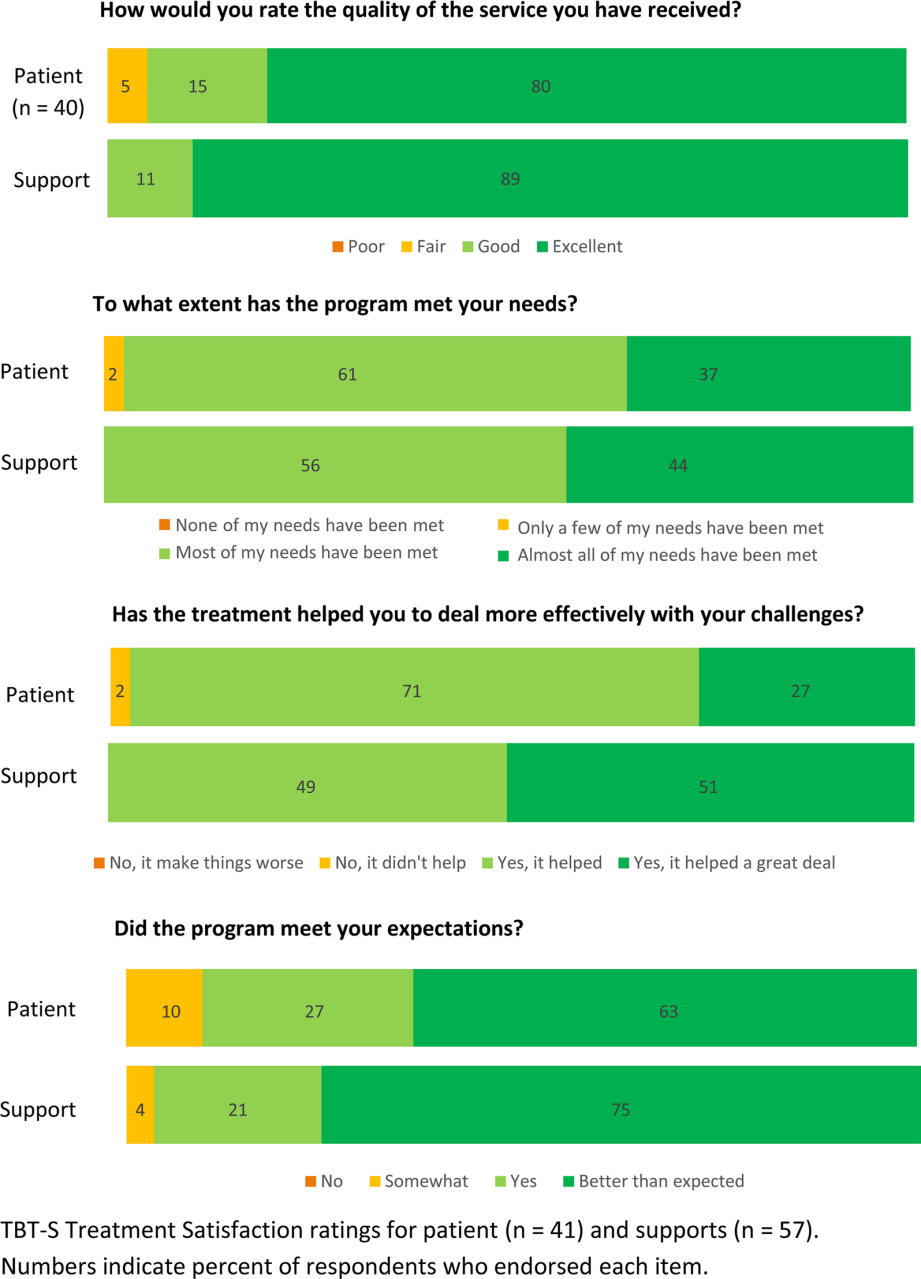
TBT-S program satisfaction questionnaire (TBT-S SQ) ratings

There were no voluntary drop-outs in any of the twelve completed TBT-S groups, nor were there any drop outs amongst the patients who opted not to participate in research (n = 4). Only one patient did not complete the treatment week, which was due to falling ill with covid-19. The 18-item PSSQ was completed by 41 patients and 57 supports (Table 2). Overall, both groups were highly satisfied with the treatment, with the supports reporting slightly greater satisfaction with the treatment, with a mean score of 4.3 for patients and 4.5 for supports. Scores on the TBT-S Comp ranged from 6.5 to 9.6 for patients, and from 7.1 to 9.8 for supports (Fig. 1). Most positively evaluated by both patients and supports were *the expertise of the clinical team* (patients mean = 9.6; supports mean = 9.8) and *the full TBT-S program* (patients mean = 9.3; supports mean = 9.7). Both patients and supports rated *eating meals together* the lowest (patients mean = 6.5; supports mean 7.1). Finally, the four TBT-S SQ items revealed high treatment satisfaction for both patients and supports (Fig. 2). All participants—both patients and supports—reported that the treatment helped them deal more effectively with their challenges, and that all or most of their needs had been met. Ninety-five percent of the patients rated the quality of the service as good or excellent, whereas 100% of the supports did the same. Lastly, the majority of participants (90% of patients and 96% of supports) reported that the program either met or exceeded their expectations.

**Table 2:** Patient and support satisfaction questionnaire (PSSQ) post treatment ratings

	Patient	Support
	(n = 41)	(n = 57)
1. I would recommend the 5-day program to others	4.8	5.0
2. I would prefer additional group treatment sessions or exercises	3.6	3.5
3. I would be willing to participate in additional group treatment sessions or exercises	4.3	4.4
4. I enjoyed the learning about the neurobiology of eating disorders through the group exercises (e.g., non-dominant hand writing exercise, brain wave)	4.6	4.8
5. The exercises on neurobiology improved my understanding about my eating disorder	4.6	4.8
6. I enjoyed the activities for learning/practicing effective communication with my Support(s)/loved one	4.4	4.7
7. I feel that my Support(s) are equipped with more/better tools for supporting me through recovery/I feel that I am equipped with more/better tools for supporting my loved one through recovery	4.6	4.8
8. I feel that I am better able to communicate with my Support(s)/loved one about my/her eating disorder	4.3	4.6
9. I enjoyed working on developing a contract/treatment plan with my Support(s)/loved one	4.2	4.6
10. I am more confident about my Support(s)'/my ability to support me/my loved one through recovery	4.3	4.6
11. I feel that my Support(s)'/my role in my/my loved one's treatment has been clarified	4.4	4.5
12. My relationship with my Support(s)/loved one has improved as a result of this treatment	4.2	4.1
13. I believe my experience from this treatment will be helpful in decreasing the likelihood that I/ my loved one will engage in behaviors such as restricting, over-exercising, purging, etc	4.0	4.3
14. I believe this treatment will be helpful in either decreasing my/my loved one's anxiety and/or other negative emotions or improving my/my loved one's ability to cope with these emotions	3.9	4.3
15. I enjoyed interacting with other patients and their Supports in the group	4.6	4.7
16. I learned skills and ideas from the other Supports and patients that I can now apply to myself/to working with my loved one in treatment	4.1	4.3
17. I felt supported by the other group members	4.4	4.7
18. I plan to continue to have my Support(s) involved in my treatment/I plan to continue my involvement in my loved one's treatment	4.6	4.9
**Mean (SD)**	**4.3 (0.3)**	**4.5 (0.4)**

### Within-subject changes in clinical measure

Table 3 shows patient and support clinical measures, including supports’ rating of patients’ eating disorder psychopathology (PEDE-Q). Immediately after the 5-day treatment, there was a significant decrease in patients’ self-reported eating disorder psychopathology (global EDE-Q) (*p* < 0.001, d = 0.77, 95% CI 0.40–1.14), and a tendency for significant decrease in support’s rating of patient’s eating disorder psychopathology (*p* = 0.054, d = 0.29, 95% CI − 0.06–0.63), with a significant decrease on the Eating Concern PEDE-Q subscale (*p* = 0.013 d = 0.41, 95% CI 0.05–0.76). There was also a significant decrease in patient’s psychosocial impairment (CIA) (*p* < 0.001, d = 0.57, 95% CI 0.21–0.92) and state anxiety (STAI State) (*p* < 0.001, d = 0.78, 95% CI 0.40–1.16) from before to immediately after the treatment, while patients’ trait anxiety remained unchanged (*p* = 0.16, d = 0.17, 95% CI − 0.16–0.51). Patients also reported significantly improved social relationships on the WHOQoL (*p* < 0.045, d = − 0.30, 95% CI 0.63–0.05). Supports reported a significant increase in (own) psychological health, as measured by one of the four subscales of WHOQOL (p = 0.16, d = 0.17, 95% CI = − 0.16–0.51). There were no differences in family functioning (FAD) as reported by patients and supports.

**Table 3:** Patient and support clinical outcomes pre- and post-treatment

	n	Pretreatment mean (SD)	Posttreatment mean (SD)	p	Cohen’ s *d*	95% CI
**PATIENTS**^*a*^						
BMI (kg/m^2^)						
Anorexia nervosa (AN)	12	16.5 (0.7)	16.6 (0.7)	**0.017**	− 0.70	− 1.32, − 0.50
Atypical AN	20	19.8 (1.2)	19.7 (1.1)	0.262	0.15	− 0.30, 0.58
EDE-Q Global	37	4.0 (1.2)	3.4 (1.3)	**< 0.001**	0.77	0.40, 1.14
* Restraint*	37	3.8 (1.5)	2.7 (1.7)	**< 0.001**	0.82	0.44, 1.19
* Eating concern*	37	3.5 (1.3)	2.7 (1.3)	**< 0.001**	0.75	0.38, 1.11
* Shape Concern*	37	4.7 (1.3)	4.4 (1.4)	**0.007**	0.42	0.08, 0.76
* AN*	11	3.9 (1.5)	3.9 (1.5)	0.302	0.16	− 0.44, 0.75
* Atypical AN*	20	4.8 (1.0)	4.4 (1.5)	**0.017**	0.51	0.04, 0.97
* Weight Concern*	37	4.1 (1.4)	3.7 (1.5)	**0.008**	0.42	0.08, 0.75
* AN*	11	3.1 (1.4)	3.0 (1.0)	0.410	0.07	− 0.52, 0.66
* Atypical AN*	20	4.3 (1.3)	3.7 (1.6)	**0.008**	0.59	0.11, 1.06
STAI state	35	58.1 (11.3)	50.3 (12.9)	**< 0.001**	0.78	0.40, 1.16
STAI trait	35	61.0 (7.6)	60.1 (8.1)	0.16	0.17	− 0.16, 0.51
WHOQOL						
* Physical health*	35	12.9 (2.3)	13.1 (2.5)	0.154	− 0.16	− 0.51, 0.16
* Psychological health*	35	9.2 (2.7)	9.5 (2.7)	0.148	− 0.18	− 0.51, 0.16
* Social relationships*	35	12.7 (3.3)	13.3 (3.2)	**0.045**	− 0.30	− 0.63, 0.05
* Environment*	35	14.5 (2.5)	14.8 (2.2)	0.166	− 0.17	− 0.50, 0.17
CIA global score	35	34.5 (9.2)	30.1 (10.5)	**< 0.001**	0.57	0.21, 0.92
FAD general functioning	36	1.9 (0.6)	1.8 (0.5)	0.059	0.27	0.07, 0.60
**SUPPORTS**						
PEDE-Q Global^b^	33	3.1 (1.2)	2.7 (1.5)	0.054	0.29	− 0.06, 0.63
* Restraint*	32	2.4 (1.6)	1.9 (1.7)	0.113	0.22	− 0.12, 0–56
* Eating concern*	32	2.9 (1.6)	2.2 (1.5)	**0.013**	0.41	0.05, 0.76
* Shape concern*	32	3.9 (1.4)	3.8 (1.8)	0.334	0.08	− 0.27, 0.42
* Weight concern*	32	3.4 (1.2)	3.2 (1.6)	0.168	0.17	− 0.18, 0.52
WHOQOL						
* Physical health*	47	15.3 (2.8)	15.4 (2.5)	0.270	− 0.09	− 0.38, 0.20
* Psychological health*	47	14.3 (1.8)	14.8 (2.0)	**0.010**	− 0.35	− 0.64, − 0.05
* Social relationships*	47	14.4 (2.5)	14.4 (2.4)	0.446	− 0.02	− 0.31, − 0.27
* Environment*	47	16.3 (2.2)	16.3 (2.2)	0.313	0.07	− 0.22, 0.37
FAD general functioning	47	1.9 (0.4)	1.8 (0.4)	0.199	0.13	− 0.16, 0.41

*Note: SD* standard deviation, *BMI* body mass index, *CIA* clinical impairment assessment, *EDE-Q* eating disorders examination questionnaire, *FAD* McMaster family assessment device, *PEDE-Q* eating disorder examination-questionnaire (Parent modified), *STAI* state trait anxiety inventory, *WHOQOL* the World Health Organization quality of life—BREF, p < 0.05 in bold

^a^Except for BMI, and EDE-Q shape-and weight concern, numbers represent the mean value across all diagnoses

^b^Weighted support data so each patient (n = 33) is equally represented

## Discussion

The current study explored feasibility, acceptability, and short-term treatment outcomes of a newly developed 5-day treatment for adults with eating disorders. Current evidence of acceptability for TBT-S is limited. To our knowledge, this is the first study testing feasibility, acceptability and treatment satisfaction of the full TBT-S treatment program, in a health-care system outside of the USA. It is also the first study to be based on the newly published TBT-S manual [7] and is an important step in assessing the potential benefits of this novel intervention. Our results indicate that TBT-S is a feasible treatment with high client satisfaction. Preliminary outcome data are also promising, and in line with two previous studies [10, 11]. However, the period between assessments is too short to make any firm conclusions, and future studies will have to follow patients over time to provide further evidence of the potential benefits for long-term outcomes.

In terms of treatment satisfaction, our findings indicate that TBT-S appealed to both patients and their supports. These results are in concordance with previous studies which have shown that TBT-S is a highly acceptable treatment [8, 11]. To obtain detailed user feedback on acceptability and treatment satisfaction, three different questionnaires were used. The PSSQ assesses patient and support satisfaction with the full TBS-program, while the TBT-S Comp evaluates the different treatment modules—whereas the TBT-S SQ reflects overall treatment satisfaction. Both patients and supports reported high satisfaction with the treatment, and would recommend it to peers in a similar situation.

Interestingly, the findings on the PSSQ are close to identical to the results by Wierenga and colleagues [11] who reported overall PSSQ mean scores that were only 0.1 above the scores of the present study, for patients as well as supports. This could indicate good cross-cultural adaptability of TBT-S. Only question number 2 on the PSSQ (*I would prefer additional group treatment sessions or exercises*) received a mean score lower than four (3.6 for patients, and 3.5 for supports). Again, these results correspond to the findings by Wierenga and colleagues (mean score 3.6 for patients, 3.9 for supports [11]) and is most likely a reflection of the participants’ satisfaction with the number of treatment sessions and/or exercises. I.e. they are content with the treatment volume.

The supports rated the different components of TBT-S slightly higher in terms of usefulness, compared to the patients’ ratings (TBT-S Comp, Fig. 1). However, both groups rated TBT-S as a whole, the expertise of the clinical team, and developing a behavioural agreement as particularly helpful elements. Even though the overall evaluation of TBT-S was positive, there were some components of the treatment which might need further consideration. Firstly, it is interesting that both patients and supports experience *eating together* as one of the least useful components of the treatment. During the TBT-S week, all participants are expected to eat breakfast, lunch and an afternoon snack at the clinic. On the first day of the treatment week (Monday), meals are provided by the clinic. Whereas on the following days, the participants bring—and prepare—their own food. Members of the clinical team join the participants during mealtimes, and provide support when needed. It is well known that mealtimes are considered one of the most difficult situations for patients with AN [29]. Thus, the lower scores on this component of TBT-S could reflect some of the challenges experienced with meals and eating for these individuals. It is also possible that the participants did not experience enough support, tools or guidance from the clinical team during meal-times, and therefore perceived this element of the treatment as less useful. In addition, our patients were in differing stages of recovery, which could contribute to the varying usefulness of eating together with other participants and/or the clinical team. Since mealtimes and providing mealtime support can be a challenging aspect of eating disorder recovery, this will be an important topic to assess further in future studies of TBT-S—with a goal of increasing the usefulness of this component. Secondly, some of the patients reviewed the dietary groups as somewhat less useful than other components of the TBT-S program. Dieticians are an integral part of the TBT-S treatment team and are present during most components of the TBT-S week. Hence, it should be underscored that the dietary groups are just one element of the dieticians’ role in TBT-S. Dieticians play an important part in developing meal plans with patients and supports—as well as providing support throughout the behavioural agreement work. The participants (both patients and supports) rate *working with dietician* and *developing a behavioural agreement* very highly. Hence, it could be that the more lecture-based approach of the dietary groups is less well received by participants, and this should be examined in future studies. Possibly by exploring the participants’ qualitative feedback on these components.

Our evaluation of overall user experience demonstrated high acceptability. The high usefulness ratings from the supports highlight an important element of TBT-S, namely including supports in treatment of eating disorders. Less than half of the supports reported that they had previously been included in treatment and 65% reported that they wished they had been more involved in previous treatments. These findings emphasise a gap in eating disorder treatments for adults, where the potential aid and support from close family and friends is often underutilized. Future studies should aim to more specifically assess in what way, and to what extent, supports can be included in the treatment of adults with eating disorders, and how clinical programs can be adapted to include supports in cases where this can be considered helpful for treatment progression.

As can be seen in the description of the patient sample (Table 1), we included a diverse range of participants in terms of age, illness severity and duration as well as current level of care. This could be viewed as a reflection of the adaptability and usefulness of TBT-S for a wide array of patients suffering from AN or other eating disorders. Since TBT-S is built from modules, the clinical team can adapt the program to meet individual needs. This is a great strength of TBT-S, and is in line with the increasing focus on personalised healthcare [30, 31]. However, as a consequence, it can make it difficult for studies to pin-point and examine the potential effective elements of TBT-S, and to what extent the results and acceptability of the model is dependent on experience of the clinical team providing the treatment. The TBT-S clinical team at RASP consists of highly specialised eating disorder therapists with a combined experience from ED treatment of over 60 years. This could explain the positive scores on the therapists’ ratings, but it also highlights the necessity for more studies of TBT-S to assess its adaptability for varying treatment settings.

Post-treatment assessments revealed significantly reduced eating disorder psychopathology and psychosocial impairment, as well as reduced state anxiety, improved social relationships and a trend towards improved family function. Since the participants eat two of their daily meals during the treatment day, it is perhaps not surprising that there is a reduction in eating disorders symptoms during the treatment week. However, it is nevertheless noteworthy that scores on weight and shape concern were also reduced, since this could indicate that some of the preoccupation with weight and shape is lowered. Interestingly, patients’ state anxiety was also significantly reduced at end of treatment while trait anxiety remained stable. This is comparable to the STAI results reported by Wierenga and colleagues [11]. A major aim of TBT-S is learning tools which can aid in coping with anxiety. Thus, it is possible that the reduction in state anxiety could reflect improved anxiety management skills at end of treatment. Knatz-Peck and colleagues [10] also reported that trait anxiety reduced over a 12-month period after completing TBT-S. However, they did not report on state anxiety. Hence, long-term follow-up studies are needed to provide any indication of the duration of the reported reduction in state anxiety.

In terms of treatment attrition, we were sensitive to the fact that elements like intensity of the treatment, practicalities of attending full day sessions as well as travel cost and time could influence treatment adherence. In addition, previous studies have shown that ambivalence towards treatment and drop-out is common for patients with AN [32]. We were therefore pleased to note that there were no voluntarily drop-outs in the current study. This is in line with previous studies of TBT-S, where attrition levels have ranged from 1.8% (n = 1; [11]) to 5.3% (n = 2; [10]). The low attrition rates of TBT-S could be attributed to the intensive format spanning five consecutive days. It is also probable that the flexibility of the program and individual tailoring to patients' needs aid in preventing drop-outs. Further, involving supports, who experienced TBT-S as highly useful, has a potential protective function in terms of attrition by encouraging their loved one to complete the program.

The current study has some important limitations to note, including a small sample size, uncontrolled design and un-blinded assessments. Thus, any post-treatment effects must be interpreted with caution and as estimates. Further, there is also a possibility that the participants were particularly motivated since the results are based on an open trial with patients (and supports) who have voluntarily applied to participate in TBT-S, thinking it will be a good fit for their challenges. Future studies should assess TBT-S efficacy in appropriately powered randomized control trials. This would allow for more accurate assessments of treatment effects. The study also has several strengths. It is the first assessment of TBT-S in a different country and health care system than its origins. Importantly, it is also the first exploration of the feasibility and acceptability of TBT-S when delivered by a clinical team other than the treatment creators. The study has good ecological validity as it included a diverse group of patients with eating disorders—and representative of the patient population which is referred to our tertiary eating disorders treatment service. This allowed us to test the feasibility for a mixed eating disorder population, and provides a reflection of how TBT-S is adaptable to individual needs. Despite a short time interval between assessments, our findings were based on reliable, validated tools which enable comparison across previous and future studies of TBT-S. In addition, by using multiple instruments of treatment satisfaction we were able to get a broad assessment of participants’ evaluation of TBT-S.

## Conclusions

This study provides a valuable contribution to the evidence-base for future research to evaluate TBT-S. Based on the current findings, TBT-S is a promising new treatment for eating disorders with high acceptability scores. Follow-up data of treatment efficacy and relapse rates will be an important avenue for future studies of TBT-S. In addition, future studies should aim to further explore methods which can most appropriately measure the effect of TBT-S and the usefulness of the different components of this treatment. Finally, randomized controlled trials across different health care systems will be necessary to assess treatment efficacy of TBT-S.

### Supplementary Information

**Additional file 1.** Temperament Based Therapy with Support (TBT-S) Overview.

## Data Availability

The datasets used and/or analysed during the current study are not publicly available due to the sensitivity of the data, but can be made available from the corresponding author on reasonable request.
