# Supplementary material for: Acceptability, feasibility and short-term outcomes of temperament based therapy with support (TBT-S): a novel 5-day treatment for eating disorders
Source: J Eat Disord. 2023 Sep 14;11:156. doi: 10.1186/s40337-023-00878-w (PMC10500782; doi:10.1186/s40337-023-00878-w)
Supplement: Supplementary file 1 — Additional file 1. Temperament Based Therapy with Support (TBT-S) Overview. [file 40337_2023_878_MOESM1_ESM.pdf]

Chapter 1

What Is Temperament Based Therapy with Support (TBT-S)?

Why TBT-S Began by Focusing on Anorexia Nervosa

Anorexia nervosa (AN) is a serious, life-threatening condition that has one of the highest death rates of all mental illnesses.[1–4] It is diagnostically defined as having extremely low body weight, an intense fear of weight gain, and disturbance in how one's body weight and shape are experienced.[5] It occurs primarily in females,[6] usually taking form during puberty with the potential to develop acute and chronic impairment over one's lifetime.[7] The illness places a considerable strain on families, friends, and work settings. While progress has been made in understanding the psychosocial and behavioral mechanisms responsible for the development and maintenance of the illness, there is an urgent need to optimize treatment approaches to reduce chronicity and improve outcomes.[7] This necessitates new and innovative treatments that contribute to long-term symptom reduction and incorporate contemporary neurobiological findings such as those covered in TBT-S.

Key Point: TBT-S has focused on AN because of its high mortality rate and low treatment efficacy over time.

TBT-S Description

Temperament Based Therapy with Support (TBT-S) is an emerging neurobiologically informed treatment approach designed to augment existing treatments. This book describes how and why TBT-S has been developed for adults with AN, recognizing it has the capacity to be applied to other psychological disorders. TBT-S fills the gap between research and clinical practice by acknowledging and treating underlying brain-based factors. TBT-S recognizes that there is a biological basis to psychological illnesses that involves temperament and altered brain function. This affects the regulation of eating and emotion for those with AN. TBT-S applies neurobiological research findings to inform treatment targets.

TBT-S combines psychoeducation and experiential activities that emphasize the key role of neurobiological factors in the development and maintenance of AN to increase insight and recognize temperament patterns. Skills-based training is used to teach clients and Supports age-appropriate strategies to manage these neurobiological factors to reduce problem thoughts and behaviors. TBT-S treatment targets include common AN temperament traits (e.g., anxiety, cognitive inflexibility, harm avoidance) and related brain processes such as altered reward and punishment sensitivity, interoception, inhibitory control, and decision-making.

https://doi.org/10.1017/9781009032063.004 Published online by Cambridge University Press

The focus of TBT-S is to work *with* clients who have AN *and* their Support persons to acknowledge, understand, and utilize their own temperament as a primary source for strength and change. Supports can include members of one's family of origin (such as parents, siblings, grandparents, adult children) and of one's "family" of choice (such as spouses, partners, friends, housemates, or colleagues). The TBT-S approach was developed and refined over a 10-year period, integrating AN research with ongoing client and Support feedback to assure the intervention strategies accurately reflect client experiences and temperaments.[8, 9] TBT-S was originally developed and studied in an intensive 5-day, 40-hour program with groups of clients and their Supports.[9]

Key Point: Temperament Based Therapy with Support (TBT-S) is an emerging and novel neurobiologically based treatment that works *with* clients' temperament to motivate change and to manage and reduce symptoms.

What Is Temperament, and Why Is It Important?

Temperament is the biological basis of our personality, influenced by genetics, brain circuit development and function over one's life span. *Character* is the external shaping of one's temperament. Temperament is to nature as character is to nurture (Figure 1.1).

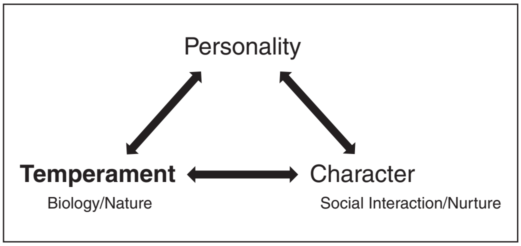

Figure 1.1 Temperament in relation to personality and character
Source: Cloninger (2014).

Temperament has been studied for more than 70 years with a primary focus on children.[10] In the late 1980s Chess and Thomas led interventions for parents and educators to incorporate children's "reactive patterns" into classroom and parenting responses.[11] They countered the trend that children were solely "products of the environment," advocating that they bring their own tendencies to the picture. Subsequently temperament researchers began to acknowledge that traits can be disadvantageous in one situation and advantageous in others.[11] Educational and parental interventions encouraged a "goodness of fit" framework for children.[12] However, therapeutic interventions that focus on shaping adults' "natural" thoughts, feelings, and behaviors have been left off the therapeutic table in the area of eating disorders.[13, 14] Working *with* clients' temperaments acknowledges who they are and what they bring of themselves to the therapeutic experience (Figure 1.2).

https://doi.org/10.1017/9781009032063.004 Published online by Cambridge University Press

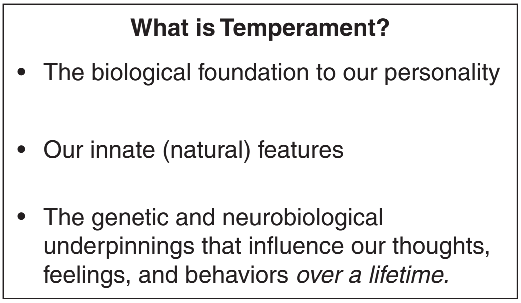

Figure 1.2 What is temperament?
Source: Cole (2020); Mitchell (2018).

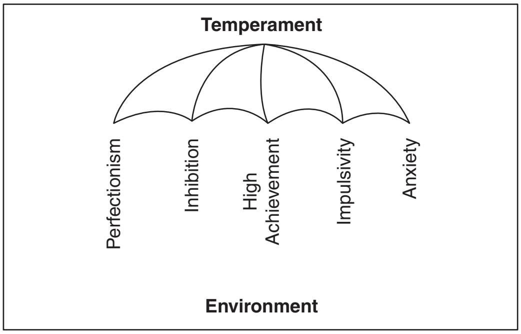

Figure 1.3 Traits are distinguishing *features* of temperament that interact with environment

Temperament is expressed through traits, as shown in Figure 1.3, which affect our thoughts, feelings, and behaviors. Temperament traits are observed in infancy and are relatively preserved across the lifespan, suggesting that personality is hardwired and consistent across life. Importantly, temperament is strongly related to most psychopathologies, especially those involving anxiety and mood disturbance. People have varying levels of vulnerabilities to develop a psychological disorder based on the traits they inherit. In fact, growing evidence indicates that temperament traits are uniquely associated with specific brain systems linked to various psychopathologies, including those involved in eating disorders (ED).[15–19] This suggests that temperament traits are genetic and brain based and have a powerful influence on ED and thus should be included in treatment approaches. See Figure 1.4.

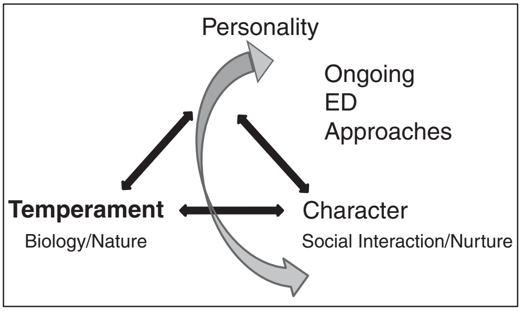

Figure 1.4 Current ED therapies focus on personality and character

https://doi.org/10.1017/9781009032063.004 Published online by Cambridge University Press

Key Point: Temperament is the biological foundation of personality and consists of traits that are fundamental expressions of each person.

Accumulating behavioral and neuroimaging evidence points to a neurobiologically based AN temperament that increases risk and contributes to the development and maintenance of the disorder.[20–36] This AN temperament is characterized by anxiety, altered sensitivity to reward and punishment, altered interoceptive awareness, difficulty with decision-making, and cognitive inflexibility and rigidity.[30, 32, 37–42] Individuals with AN also tend to be high achieving, perfectionistic, inhibited, and rule abiding. These temperament and personality traits are related to altered insula and fronto-striatal neural circuit function, highlighting their neurobiological basis.[43–47] In addition to predating the disease, these traits often persist in a mild to modest degree after recovery, offering evidence they are biologically based traits and not behavioral symptom expressions.[30, 37–40, 48]

This AN temperament profile serves as a framework that identifies the neurobiological constructs and traits targeted in TBT-S; and guides the interventions designed to address symptoms specific to AN. TBT-S has been developed to fill a gap in ED treatment (Figure 1.5). It features AN temperament, focusing on the traits that make a person vulnerable to AN, why AN symptoms emerge and are maintained, and how to shift trait expressions or construct environmental modifications to reduce symptoms to impact positive change. See Figure 1.5.

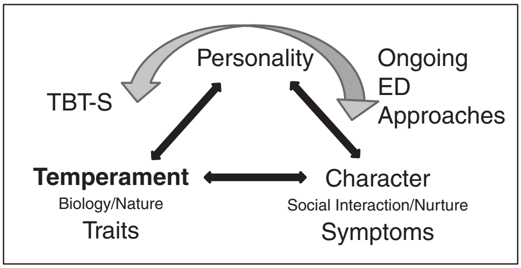

Figure 1.5 TBT-S augments other treatments by focusing on temperament

Key Point: AN has common traits creating a temperament profile that guides TBT-S interventions to work *with* client traits as natural resources for lifelong changes.

The theoretical model of TBT-S approaches the illness from the inside out. It begins with genes. Environmental factors (such as life stresses, malnutrition, and trauma) can modify gene expression and temperament via epigenetic processes (e.g., gene × environment interactions), suggesting that although traits are relatively stable, they can be shaped by experience and treatment.[49] Figure 1.6 shows the relationship between temperament and environmental influences on symptoms. Genes, the center circle, code how the brain wires its circuits impacting thoughts, perceptions, feelings, and actions. Traits develop from brain circuits structured by genes which are influenced by the environment. Persons with AN have specific alterations in the wiring of these brain circuits that contribute to destructive AN trait expressions. Other circuits function normally, affording healthy and/or above

https://doi.org/10.1017/9781009032063.004 Published online by Cambridge University Press

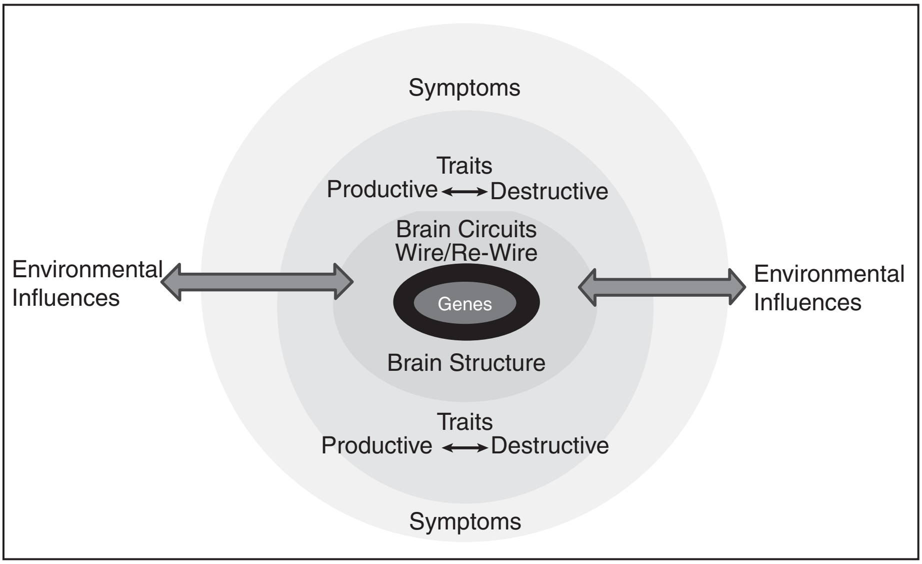

Figure 1.6 The relationship between traits and environmental influences on symptoms

average productive trait expressions. Altered trait expressions impact the type of symptoms that develop. For example, elevated harm avoidance is related to dietary restriction and reduced social interactions. TBT-S focuses on acknowledging and utilizing one's trait expressions to modify, shape, and promote recovery. For example, TBT-S prescribes structure and routine, in alliance with a rule-bound trait, to reduce anxiety and intolerance of uncertainty around meals. The inside-out approach of TBT-S complements ongoing therapies that are grounded in environmental and behavioral models.

Key Point: The TBT-S theoretical model approaches treatment from the inside out, targeting traits and underlying biology that complements other treatment approaches which work from the outside in.

Who Are the Intended Participants?

TBT-S was designed to be administered to adult clients and their Supports. Thus, both clients and Supports participate with the clinical team. Conducting TBT-S with clients and their Supports is a powerful and effective way to ensure information is relayed, experienced, and processed similarly. We have focused our research and this book on adults with AN and their broad range of Supports in response to the higher chronicity and mortality within this subpopulation. The TBT-S focus is on improving client-Support relationships and communication to facilitate recovery. See Figure 1.7.

https://doi.org/10.1017/9781009032063.004 Published online by Cambridge University Press

TBT-S is a treatment approach that:

- Works *with* clients' nature, their temperament/traits
- Explains the neurobiological underpinnings of client trait expressions
- Draws on assistance from Supports for clients of *all ages*.

Figure 1.7 Temperament Based Therapy with Support (TBT-S)

Key Point: TBT-S endorses that Supports are included in the treatment of adults with anorexia nervosa to increase understanding of the illness and improve communication and continuity in care.

Where Can TBT-S Be Applied?

TBT-S core principles and modules could be applied in (a) one-on-one, (b) client with Support person, and(c) client and/or Support group sessions either (d) virtually or face-to-face, (e) ranging from outpatient through day hospital levels of care. When TBT-S core principles are described in therapy sessions, clients learn about the neurobiological underpinnings of their temperament expressions and skills to help shape their traits. The therapeutic change process is enhanced when additional components include virtual or face-to-face: (a) Support person(s), and (b) the act of practicing the skills together. The learning and change process can be further enhanced by providing TBT-S interventions in group settings through the power of client and Support interactions.[183]

TBT-S was originally developed in a 5-day group intensive format to maximize efficacy, build on skill development intensively, minimize attrition, and provide a practical and accessible approach for adult clients and Supports to work together in a discrete amount of time. Increased treatment frequency and intensity are critical components needed to elicit behavior change.[50, 51] Treatment models for anxiety indicate that intense, repeated, and focused *in vivo* practice is key to altering biologically driven avoidance behaviors by maximizing learning through massed practice and allowing close monitoring of compliance.[52–59]

Intensive treatment formats also show an increase in initial efficacy.[60, 61] This appears to be critical in treating AN, as evidence suggests that adolescents with AN who gain weight in the first four sessions of family-based treatment (FBT) have significantly better outcomes than those who do not gain weight early in treatment.[62] An intensive treatment format offers several additional advantages, including reduced burden on the adult client and Support(s) of having to commit to treatment over long periods of time, thus likely improving accessibility, acceptability, and compliance. An initial intensive model may also be a cost-effective "jump-start" to shorten residential or partial hospital treatments and augment outpatient ED treatments.

In the studied TBT-S 5-day model, multiple adult clients and Supports are treated together in a group structure, commonly known as a multi-family group in adolescent

https://doi.org/10.1017/9781009032063.004 Published online by Cambridge University Press

treatment. Group settings enable and facilitate peer-to-peer consultation, a powerful method of learning that can improve outcomes. Having multiple adult clients and their Supports together can improve participants' understanding of the illness by allowing them to learn from similar and diverse perspectives during group activities. Working with groups of clients and Supports broadens viewpoints on effective ways to manage recovery and generates new ideas. The TBT-S focus is on improving client-Support relationships and communication to facilitate recovery.

Key Point: TBT-S was studied in a 5-day group format to intensify treatment intervention, unify clients and Supports, and increase accessibility to treatment.

TBT-S Modular Structure

TBT-S is implemented as a modular treatment, meaning that it has multiple, individual, complementary treatment interventions that have been studied in different combinations as part of the 5-day TBT-S program. TBT-S treatment modules were designed to target symptoms using a broad array of intervention strategies (e.g., psychoeducation, experiential learning, skills training, meal coaching and behavioral agreements) that include both the client and Supports. Each TBT-S treatment module is described in Section 3 and includes a variety of treatment activities. Clinicians can select activities from each module deemed to be most relevant depending on the clinical presentations and developmental stage of the clients and their Supports present in treatment.

Many clinicians may not have the infrastructure to deliver TBT-S in its tested 5-day format. Individual treatment modules or activities can be administered independently across multiple treatment settings, including outpatient through partial hospitalization settings, and in multiple formats, such as group, individual, or family therapy settings (See Section 4). The TBT-S multi-day design has also been administered in shorter intensive formats over 2 to 4 days in outpatient and higher level of care settings in the United States, Canada, Norway and Greece. Efforts to deliver the multi-day TBT-S program in a virtual format also appear promising. Section 4 of this manual provides examples of how to structure and organize modules by treatment target or temperament trait in multiple treatment settings.

Key Point: Clinicians can flexibly apply TBT-S integrating its modular format with multiple treatment strategies to formulate unique stylized interventions.

Two TBT-S Versions: Young Adult (YA) and Severe-and-Enduring Anorexia Nervosa (SE-AN)

Two versions of TBT-S have been developed and tested to date, YA and SE-AN. Both versions uphold TBT-S core principles (see Chapter 2) as central themes and adhere to the same structure, format of treatment and the level of Support involvement. They differ in how adults are approached when in young adult stages of life development compared to adults who have severe-and-enduring forms of AN.

https://doi.org/10.1017/9781009032063.004 Published online by Cambridge University Press

Young Adult TBT-S (YA TBT-S)

The Young Adult model of TBT-S (YA TBT-S) is designed for clients with AN and other restrictive-type ED between the ages of 17 and 27 and their parents, who are automatically nominated as primary Supports. This highly focused version of TBT-S was designed to enhance treatment by integrating important developmental considerations since many adults seeking treatment for AN often fall within this age range. Naturally, young adults with AN are undergoing developmental changes and growth that are central to this age and often have a primary impact on treatment and recovery, both individually and within the context of their family system. YA TBT-S is tailored to provide education on neurobiology, skills training, and a model of family assistance that takes into account these important developmental considerations.

Emerging adulthood is a developmental window that has gained more attention in recent years, in part because the incidence of mental illness is highest in this developmental stage. Young adults do not squarely fit into either adult or child/adolescent services because most in this age category are embarking on the launch to independence (versus being fully independent) and, increasingly so, continue to be embedded in their family system in important ways. Young adults are adjusting to significant life transitions during this developmental stage, including separation from family of origin, increasing autonomy and individual responsibility, and more commonly in this modern era, interdependence within their family of origin.

Developmentally, young adults are striving to individuate from their family, learning to make their own decisions, and increasingly focusing on carving their own path in life. This developmental backdrop is significant and imposing in the life of a young adult and thus deserves consideration when working with clients with AN in this age range. YA TBT-S brings these developmental dilemmas to the forefront of treatment and addresses them within the context of recovery and treatment. Themes touched on in treatment include the capacity for change within the context of the temperament framework due to neurodevelopment, how to assist a YA in recovery from AN, how to make use of continued parental involvement to positively impact recovery, how to strike a balance of autonomous and family-focused recovery and navigating an effective working relationship among YAs and their parents.

Severe-and-Enduring Anorexia Nervosa TBT-S (SE-AN TBT-S)

The SE-AN version of TBT-S is designed for all adults with AN across the life span who have chronic AN. It recognizes that traditional adult therapy focuses on individuation. In contrast, TBT-S integrates interdependence within a neurobiological framework. In this model, Supports may be anyone the client designates as a “support person.” The SE-AN version of TBT-S has been studied with clients of all ages, from newly graduated 18-year-old females and males to older clients in their 50s and 60s who have developed chronic AN tendencies. As SE-AN clients age, they tend to turn to a wider diversity of Support persons, with many of their primary family members having “burned out.” Trait expressions continue throughout one’s lifetime. When TBT-S is offered in a group format, the expertise from older clients offers lived wisdom informing younger clients of symptoms that can become ingrained over time, while the younger clients resurrect hope and motivation in the older clients to reshape trait expressions. TBT-S presents neurobiological information that is congruent with AN temperament, increasing awareness that there are tools that those with AN can utilize that align with their own temperament.

https://doi.org/10.1017/9781009032063.004 Published online by Cambridge University Press

Clients tend to enter treatment assuming they have little within themselves that can be a part of the solution. They report having tried many behavioral interventions, but some have failed over time. In a TBT-S approach, the adult client educates, coaches, and clarifies with their Supports their own experiences of what it is like to have AN traits, how symptoms allow relief and lower anxiety, and what helps and does not help. Clients actively explore how to utilize the same traits that have been expressed destructively in more productive ways. The discovery that their authentic selves, that their own temperament, has worth and is a means toward health and well-being motivates change and empowers their strengths. The more SE-AN adult clients align with the biological bases of their personality, the sooner they can begin to identify how to use their own traits to impact change. SE-AN adults have engrained rule-bound rituals that have sustained their ED symptoms over years. TBT-S utilizes its trait-based approach to empower the rule-bound traits as solutions to step away from destructive tendencies, using tools to shift the same traits toward productive expressions.

Key Point: There are two versions of TBT-S. YA TBT-S is designed for ages 17–27 and integrates important developmental considerations into the TBT-S model. SE-AN TBT-S is designed for ages 18–60 who have chronic AN symptoms (over five years of illness). Both versions interrupt and shift AN symptoms by teaching clients to identify *their own* trait-based behavioral solutions.

Summary Key Points

- **Temperament Based Therapy with Support (TBT-S) is an emerging neurobiologically informed treatment approach designed to augment existing treatments.**
- **TBT-S has been developed for adults with anorexia nervosa (AN), recognizing it has the capacity to be applied to other psychological disorders.**
- **TBT-S fills the gap between research and clinical practice by acknowledging and treating underlying brain-based factors.**
- **TBT-S recognizes that there is a biological basis to psychological illnesses that involves temperament and altered brain function.**
- **It is developed as a modular treatment to be administered virtually or face-to-face. Clinicians can insert TBT-S modules into ongoing therapies ranging from one module in an outpatient treatment setting to a day of TBT-S to the 5-day, 40-hour, 1-week TBT-S program that was studied.**
- **The "S" of TBT-S means "Support," the word chosen by adults clients with AN to describe anyone who offers support.**

https://doi.org/10.1017/9781009032063.004 Published online by Cambridge University Press
